# Real-World Diagnostic Phenotypes and Treatment Pathways in Trigeminal Pain: A Retrospective Tertiary-Center Cohort—Diagnostic Phenotypes in Trigeminal Pain

**DOI:** 10.3390/neurolint18050099

**Published:** 2026-05-21

**Authors:** Shachar Zion Shemesh, Paz Kelmer, Jose Asprilla, Yotam Hadari, Omri Cohen, Lior Ungar

**Affiliations:** 1 Department of Neurosurgery, Sheba Medical Center, Ramat Gan 52621, Israel; shemesh.shachar@gmail.com (S.Z.S.); pazkelmer92@gmail.com (P.K.); asprilla_05@hotmail.com (J.A.);; 2 Gray Faculty of Medicine, Tel Aviv University, Tel Aviv 52621, Israel; 3 Dina Recanati School of Medicine, Reichman University, Herzelia 4610101, Israel; 4 Pain Center, Sheba Medical Center, Ramat Gan 52621, Israel

**Keywords:** trigeminal neuralgia, facial pain, secondary trigeminal neuralgia, multiple sclerosis, tumor-related trigeminal pain, dental misdiagnosis, MRI, microvascular decompression, radiofrequency thermocoagulation, balloon compression, stereotactic radiosurgery

## Abstract

**Background:** Trigeminal neuralgia (TN) is clinically defined, but patients presenting to tertiary practice with trigeminal-region pain are often diagnostically heterogeneous and may follow prolonged medication, dental, imaging, and procedural pathways before a stable phenotype is established. We aimed to characterize diagnostic phenotypes, secondary causes, and treatment-escalation patterns in a large retrospective tertiary-center trigeminal pain cohort derived from routine free-text clinical documentation. **Methods:** We conducted a retrospective single-center cohort study based on a clinical dataset containing 18,007 note fragments linked to 672 unique patient records between 12 October 2010 and 21 April 2026. A rule-based natural-language-processing-assisted chart review framework was used to identify patients with trigeminal pain and to extract documentation-derived demographic features, pain distribution, secondary causes, dental pathway variables, imaging signals, medication exposure, procedures, and outcome language. Patients were grouped into primary/classical TN, secondary TN/trigeminal pain, and dental-first or mimic pathways using predefined operational criteria. **Results:** A total of 455 patients met criteria for the analytic trigeminal pain cohort; 311 (68.4%) carried explicit TN terminology. Mean age was 58.7 years, median age 60 years, and 267 of 428 patients with recoverable sex data (62.4%) were women. Trigeminal branch involvement could be extracted in 351 patients (77.1%), with V2 involvement documented in 256 (56.3%), V3 involvement in 218 (47.9%), and V1 involvement in 138 (30.3%). The final NLP-derived phenotypic distribution comprised 201 primary/classical TN cases (44.2%), 146 secondary TN/trigeminal pain cases (32.1%), and 108 dental-first or mimic presentations (23.7%). MRI was documented in 384 patients (84.4%), neurovascular conflict or vascular loop in 253 (55.6%), multiple-sclerosis-related disease in 69 (15.2%), and tumor-related trigeminal involvement in 84 (18.5%). Prior dental evaluation was identified in 169 patients (37.1%), and prior dental procedures in 114 (25.1%). Carbamazepine exposure was documented in 367 patients (80.7%), pregabalin in 221 (48.6%), gabapentin in 150 (33.0%), oxcarbazepine in 116 (25.5%), and phenytoin in 73 (16.0%). At least one invasive or image-guided procedure was documented in 390 patients (85.7%), including nerve blocks/injections in 355 (78.0%), radiofrequency procedures in 126 (27.7%), balloon compression in 90 (19.8%), microvascular decompression in 113 (24.8%), and stereotactic radiosurgery in 55 (12.1%). Dental-first patients were significantly more likely to have undergone prior dental procedures (65.7% vs. 3.5% in primary/classical TN and 24.7% in secondary TN; *p* < 0.001), whereas secondary TN/trigeminal pain was associated with higher use of radiofrequency procedures (36.3%; *p* = 0.017), higher use of stereotactic radiosurgery (19.9%; *p* = 0.002), higher recurrence documentation (70.5%; *p* = 0.001), and a higher rate of complete pain relief documented at last follow-up (46.6%; *p* = 0.004). **Conclusions:** In tertiary practice, trigeminal pain is substantially broader than a formal TN label. Secondary disease and dental-first pathways account for a large fraction of referrals, and management is characterized by heavy medication burden, frequent escalation, and recurrent retreatment. A structured phenotyping approach may help convert routine clinical documentation into a clinically meaningful framework for diagnostic triage and treatment selection, although imaging and outcome variables require cautious interpretation when derived from retrospective free text.

## 1. Introduction

Trigeminal neuralgia is among the most severe pain syndromes encountered in neurology and neurosurgery. It is classically defined by unilateral, brief, electric-shock-like attacks affecting one or more divisions of the trigeminal nerve, frequently triggered by innocuous stimuli such as talking, chewing, shaving, touching the face, or brushing the teeth [1,2,3,4,5,6]. In modern classification systems, however, TN is no longer viewed as a single homogeneous entity. Contemporary frameworks distinguish classical TN, idiopathic TN, and secondary TN, and also recognize the clinically important subgroup with concomitant continuous pain between paroxysms [1,2,3,4,5,6,7]. This distinction is not purely academic. Phenotype influences differential diagnosis, imaging priorities, expected response to pharmacotherapy, and the choice and durability of invasive treatment.

Despite widely cited diagnostic criteria and international guidelines, the real-world recognition of trigeminal pain disorders remains inconsistent [3,8,9,10]. Patients may first present to primary care, dentistry, oral medicine, otolaryngology, or general neurology, and many experience prolonged diagnostic delay before the pain syndrome is clearly identified [10,11,12,13,14,15,16]. In clinical practice, classical TN often overlaps with or is initially confused for painful trigeminal neuropathy, persistent idiopathic facial pain, atypical odontalgia, temporomandibular disorders, sinus or dental pathology, post-traumatic pain, or tumor-related cranial neuropathies [8,11,14,15,16]. From a neurosurgical standpoint, such imprecision has direct consequences. Patients may undergo unnecessary dental procedures, prolonged ineffective medication trials, delayed magnetic resonance imaging (MRI), or escalation to procedures that are not optimally aligned with the underlying disease biology.

Secondary trigeminal pain warrants particular attention because it changes both the diagnostic pathway and the therapeutic logic. Multiple sclerosis and structural lesions along the trigeminal pathway, including cerebellopontine angle and skull-base tumors, can generate a TN-like phenotype while carrying distinct prognostic and management implications [17,18,19,20,21,22,23,24]. In these settings, MRI is central not only for lesion identification, but also for distinguishing clinically meaningful neurovascular conflict from incidental contact and for determining whether decompression, lesion-directed treatment, or ablative strategies are more appropriate [21,22,23,24,25,26,27,28,29]. Conversely, even in apparently classical TN, imaging must be interpreted with nuance; negative studies do not always exclude surgically relevant compression [25,26,27,28,29].

Management pathways are similarly heterogeneous. Carbamazepine and oxcarbazepine remain the foundation of first-line treatment, yet real-world care often involves pregabalin, gabapentin, baclofen, lamotrigine, phenytoin, botulinum toxin, or multidrug regimens when efficacy is incomplete or adverse effects become limiting [3,30,31,32]. Once medication fails or becomes poorly tolerated, escalation may proceed to microvascular decompression (MVD), radiofrequency thermocoagulation, percutaneous balloon compression, stereotactic radiosurgery, or repeat procedures, with treatment selection influenced by phenotype, age, comorbidity, imaging, prior intervention, and surgeon preference [28,33,34,35,36,37,38,39].

The present dataset offers a different perspective from that of traditional TN registries. Rather than beginning with a pre-curated cohort of unequivocal TN, it captures the broader population of patients reaching tertiary care with trigeminal-region pain. This makes it possible to study not only explicit TN cases, but also secondary syndromes, mixed phenotypes, and patients whose pathway initially ran through dental or other non-neurosurgical channels. We therefore designed the current study to address three aims: to characterize the phenotypic composition of a tertiary trigeminal pain cohort, to quantify the burden of secondary and dental-first pathways, and to describe real-world medication and procedural escalation across this population.

## 2. Materials and Methods

### 2.1. Study Design and Data Source

This retrospective single-center cohort study was based on a clinical dataset from a tertiary facial-pain/neurosurgical practice environment. The source file contained 18,007 note fragments linked to 672 unique patient identifiers and spanned 12 October 2010 through 21 April 2026. Notes included chief complaint, current illness, visit summaries, referral reasons, operative narratives, postoperative documentation, and related free-text clinical fields.

### 2.2. Cohort Identification

A broad trigeminal pain cohort was assembled at the patient level using a rule-based search strategy combining trigeminal terminology with supportive pain-pathway features. Identification terms: variants of trigeminal neuralgia, trigeminal pain, trigeminal neuropathy, trigeminal nerve involvement, cranial nerve V, and TN. To improve specificity, broad trigeminal terminology was paired with supportive evidence such as facial pain localization, branch notation (V1/V2/V3), neuralgic descriptors, trigger-related language, dental pathway language, or TN-directed procedural terminology.

Patients were assigned to phenotypic groups using a prespecified hierarchical decision framework. First, patients were classified as having secondary TN/trigeminal pain if the available documentation indicated multiple sclerosis or a structural lesion involving or plausibly affecting the trigeminal pathway, including the cerebellopontine angle, Meckel’s cave, skull base, posterior fossa, or other tumor-related trigeminal involvement. This secondary category took precedence over all other pathway variables; therefore, a patient with multiple sclerosis or tumor-related trigeminal involvement and a prior dental procedure remained classified as secondary TN/trigeminal pain. Second, among patients without documented secondary disease, cases were classified as dental-first or mimic pathway if the records described dental evaluation, dental procedures, odontalgia-like attribution, or referral through a dental/oral-maxillofacial pathway before or during the trigeminal pain workup. Third, the remaining patients with TN terminology or TN-supportive clinical features, without documented secondary disease or a dental-first/mimic pathway, were classified as primary/classical TN.

The dental-first or mimic category was intended to capture a real-world referral pathway rather than a single diagnostic entity. It may include patients who were ultimately felt to have TN, atypical odontalgia, persistent idiopathic facial pain, temporomandibular or dental mimics, or other non-secondary trigeminal-region pain syndromes when these distinctions could not be reliably separated from free-text documentation. Cases with postherpetic neuralgia, post-traumatic trigeminal neuropathy, or other clearly specified non-TN neuropathic diagnoses were not classified as primary/classical TN; when present without multiple sclerosis or tumor-related disease, they were retained within the broader mimic pathway.

### 2.3. Data Extraction

Extraction was performed using a rule-based natural-language-processing-assisted chart review framework applied to aggregated patient-level text. The following variables were extracted: age at index documentation, sex, branch involvement, laterality, paroxysmal and continuous pain descriptors, trigger factors, sensory abnormalities, dental pathway variables, MRI documentation, vascular conflict terminology, multiple sclerosis, tumor-related involvement, medication exposure, procedures, improvement, complete pain relief, recurrence, and adverse-effect language. Medication extraction included carbamazepine, oxcarbazepine, pregabalin, gabapentin, baclofen, phenytoin, duloxetine, and amitriptyline. Procedure extraction included nerve blocks/injections, radiofrequency procedures, balloon compression, MVD, and stereotactic radiosurgery.

### 2.4. Manual Review and Extraction Validation

The extraction framework was designed as an NLP-assisted chart review tool rather than a fully autonomous classifier. Rule outputs were reviewed at the patient level for internal consistency, duplicate note fragments, and clinically implausible combinations. Because the present study was based on retrospective routine documentation rather than a prospectively curated registry, dual-review manual adjudication with formal inter-rater agreement statistics was performed for the entire cohort. Accordingly, extracted variables should be interpreted as documentation-derived clinical signals rather than independently adjudicated endpoints.

Outcome variables required explicit documentation in the available notes. “Documented improvement” was defined as any free-text statement indicating partial or meaningful clinical improvement after medication or procedure. “Complete pain relief” required language consistent with the absence of pain, pain freedom, complete response, or equivalent phrasing at the last available documented follow-up. “Recurrence” required documentation of recurrent pain, relapse, return of symptoms, repeat treatment for recurrent symptoms, or equivalent wording after a period of improvement. These outcomes were not intended to replace standardized prospective outcome instruments such as Barrow Neurological Institute pain intensity grading, pain-free survival, or medication-free remission, which were not consistently available in the source documentation.

### 2.5. Outcome Measures

The primary study outcome was the phenotypic distribution of trigeminal pain at tertiary presentation. Secondary outcomes included documented secondary causes, dental-first pathway burden, MRI documentation, MRI-derived textual signals of neurovascular conflict, multiple sclerosis, tumor-related trigeminal involvement, medication exposure, invasive treatment use, and pathway-associated outcome signals including documented improvement, complete pain relief, and recurrence at last available follow-up. Imaging variables were extracted from clinical notes and radiology-report language when available; the study did include centralized neuroradiological re-review of MRI scans, assessment of MRI protocol quality, or systematic adjudication of whether a reported vascular contact was clinically symptomatic.

### 2.6. Statistical Analysis

Continuous variables are reported as mean ± standard deviation or median with interquartile range where appropriate; categorical variables are presented as counts and percentages. Comparisons across phenotypic groups were performed using the Kruskal–Wallis test for age and chi-square test for categorical variables. All analyses were exploratory and intended to describe real-world pathway structure rather than infer causal treatment effects. No correction for multiplicity was applied; therefore, between-group p values should be interpreted as hypothesis-generating rather than confirmatory.

## 3. Results

Of the 672 unique patients represented in the source dataset, 455 met criteria for the analytic trigeminal pain cohort. Explicit TN terminology was documented in 311 patients (68.4%). Age was recoverable in 429 patients (94.3%), with a mean age of 58.7 years and a median age of 60 years. Sex was recoverable in 428 patients, among whom 267 (62.4%) were women and 161 (37.6%) were men.

Trigeminal branch involvement could be extracted in 351 patients (77.1%). V2 was the most frequently documented division, present in 256 patients (56.3%), followed by V3 in 218 (47.9%) and V1 in 138 (30.3%). Paroxysmal neuralgic descriptors were common, identified in 293 patients (64.4%), whereas continuous or background pain descriptors were present in 287 patients (63.1%), underscoring the clinical complexity of the referral population rather than a pure classical-TN registry (Table 1).

The final NLP-derived phenotypic distribution comprised 201 primary/classical TN cases (44.2%), 146 secondary TN/trigeminal pain cases (32.1%), and 108 dental-first or mimic presentations (23.7%). Patients in the primary/classical TN group were older than those in the dental-first and secondary groups (mean age 61.0 vs. 56.9 and 57.2 years, respectively; *p* = 0.014). Explicit TN terminology was common across the cohort, but the clinically important finding was that nearly one-third of patients were classified as secondary trigeminal pain, and nearly one-quarter followed a dental-first or mimic pathway before or during tertiary evaluation.

MRI was documented in 384 patients (84.4%), whereas 71 patients (15.6%) had no MRI explicitly documented in the extracted clinical text. The absence of MRI documentation should not be interpreted as proof that imaging was not performed; in a retrospective tertiary referral dataset, this may reflect external imaging, incomplete transfer of radiology reports, historical notes outside the available fields, or documentation focused on treatment rather than diagnostic workup. Among the full cohort, neurovascular conflict or vascular-loop language was identified in 253 patients (55.6%), multiple-sclerosis-related disease in 69 (15.2%), and tumor-related trigeminal involvement in 84 (18.5%). These MRI-related categories were not mutually exclusive and were not intended to sum to 100%. For example, a patient with multiple sclerosis or tumor-related trigeminal involvement could also have vascular-contact language in the report (Table 2).

The extracted MRI variables should therefore be interpreted as documentation-derived imaging signals rather than centrally adjudicated imaging diagnoses. A negative or non-informative MRI signal in this dataset means that the available text did not contain the relevant term; it does not exclude missed neurovascular compression, inadequate MRI protocol, short or asymmetric cisternal trigeminal nerve anatomy, porus trigeminus compression, or clinically incidental vascular contact. Across phenotypes, MRI documentation did not differ significantly, but lesion-directed patterns were concentrated in the secondary group, as expected from the operational definition.

Dental pathway burden was substantial. Prior dental evaluation was identified in 169 patients (37.1%), and 114 patients (25.1%) had undergone a dental procedure, including extraction, root-canal treatment, implant-related workup, or equivalent interventions. Dental procedures were strikingly concentrated in the dental-first group, in which 71 of 108 patients (65.7%) had undergone a prior dental procedure, compared with 7 of 201 primary/classical TN patients (3.5%) and 36 of 146 secondary TN/trigeminal pain patients (24.7%; *p* < 0.001).

Medication exposure was extensive. Carbamazepine was documented in 367 patients (80.7%), pregabalin in 221 (48.6%), gabapentin in 150 (33.0%), oxcarbazepine in 116 (25.5%), phenytoin in 73 (16.0%), and baclofen in 38 (8.4%). Carbamazepine remained the dominant anchor therapy across all phenotypes, without significant between-group differences in overall exposure. Pregabalin and gabapentin were also common, supporting the impression that many patients entered tertiary care after multiple medication trials or combination therapy.

At least one invasive or image-guided procedure was documented in 390 patients (85.7%). Nerve blocks or injections were reported in 355 patients (78.0%), radiofrequency procedures in 126 (27.7%), balloon compression in 90 (19.8%), MVD in 113 (24.8%), and stereotactic radiosurgery in 55 (12.1%). Overall procedural exposure was high across all phenotypes, but radiofrequency procedures were more common in the secondary group than in the primary/classical or dental-first groups (36.3% vs. 24.4% and 22.2%, respectively; *p* = 0.017). Stereotactic radiosurgery was also clustered in the secondary group (19.9% vs. 7.5% in primary/classical TN and 10.2% in dental-first cases; *p* = 0.002). In contrast, MVD and balloon compression were used at comparable frequencies across groups.

Diagnostic nerve blocks or injections were captured as procedural exposures; however, the retrospective free-text structure did not reliably distinguish procedures performed primarily for diagnostic confirmation from those performed for therapeutic pain control. Similarly, the dataset did not support a reliable cross-tabulation of each invasive procedure by centrally adjudicated MRI-positive versus MRI-negative status. In particular, because MRI positivity was extracted from documentation rather than re-reviewed imaging, we avoided inferring whether individual MVD, radiosurgery, or percutaneous cases were performed in the presence or absence of truly symptomatic neurovascular conflict (Table 3).

Documented improvement of any degree was identified in 375 patients (82.4%), and complete pain relief at the last documented follow-up was identified in 165 (36.3%). Recurrence language was present in 283 patients (62.2%). Complete relief was most frequent in the secondary group (46.6%) and least frequent in the dental-first group (27.8%; *p* = 0.004), whereas recurrence was more common in secondary and dental-first pathways than in primary/classical TN (70.5% and 68.5% vs. 52.7%; *p* = 0.001). This counterintuitive coexistence of higher complete-relief documentation and higher recurrence documentation in the secondary group likely reflects treatment heterogeneity and repeated tertiary-care encounters rather than superior durability of treatment in secondary disease. Because the extracted dataset did not consistently separate tumor-directed surgery, radiosurgery for MS-related TN, percutaneous procedures, medication response, and lesion-directed oncologic management within this subgroup, the finding should be interpreted as an exploratory documentation-derived signal rather than a comparative efficacy result.

## 4. Discussion

This study demonstrates that a tertiary trigeminal pain practice is not simply a repository of classical TN. In our cohort, fewer than half of all patients were classified as primary/classical TN, whereas nearly one-third had secondary TN/trigeminal pain, and almost one-quarter followed a dental-first or mimic pathway. That observation is clinically important because it shifts the focus of tertiary facial-pain care away from a narrow procedure-centered model and toward a broader diagnostic triage model. In other words, the central challenge is not merely choosing among MVD, radiofrequency, balloon compression, or radiosurgery after the diagnosis is already secure. The challenge is recognizing which patients truly have classical TN, which patients have secondary disease, and which patients are being misrouted through dental or other pathways before the phenotype is clarified.

The burden of secondary disease in this cohort was substantial. Multiple sclerosis was documented in 15.2%, and tumor-related trigeminal involvement in 18.5%. These figures are higher than would be expected in an unselected community-practice population and should be interpreted as tertiary-center referral enrichment rather than population prevalence. Patients with suspected tumor-related, skull-base, cerebellopontine angle, Meckel’s cave, or multiple sclerosis-related trigeminal pain are more likely to be referred to neurosurgical and multidisciplinary facial pain services, which probably explains the high proportion of secondary cases in the present cohort. This referral bias is clinically informative but limits generalizability.

Collapsing structural lesions into a single tumor-related category is another limitation of the present analysis. The free-text dataset allowed reliable identification of tumor-related trigeminal involvement, but it did not consistently support granular tumor classification by histology, exact site, size, growth pattern, or treatment status. Consequently, the tumor-related group should be interpreted as a broad structural lesion signal rather than as a homogeneous disease category. Future prospective registries should separate vestibular schwannoma, meningioma, epidermoid tumors, Meckel’s cave lesions, skull-base metastases, and other structural causes, because these entities may have different imaging patterns, referral pathways, and treatment responses.

The association we observed between secondary disease and both radiofrequency use and stereotactic radiosurgery is biologically and clinically plausible. It suggests that, in routine practice, lesion-associated or medically complex cases are more likely to be steered toward less invasive, repeatable, or lesion-adapted strategies than toward a purely decompressive paradigm. However, because this study was not designed to compare procedure efficacy and did not include central imaging review or standardized pain-outcome grading, these treatment patterns should be considered descriptive rather than causal.

The dental pathway signal was clinically important. More than one-third of the full cohort had prior dental evaluation, and one-quarter had undergone a dental procedure. In the dental-first group, nearly two-thirds had undergone a dental procedure before or during the trigeminal pain workup. Although we initially regarded these proportions as high, they may still underestimate the dental burden observed in other health-care settings, particularly in systems where access to dentistry, oral-maxillofacial evaluation, neurology, pain medicine, and neurosurgery differs from the Israeli tertiary-referral environment. Thus, the dental pathway signal should be interpreted not only as a marker of diagnostic confusion, but also as a health-system and referral-pattern variable.

This finding supports the literature describing TN and TN-like syndromes as frequent sources of dental misdirection [11,12,13,14,15,16]. For the practicing neurosurgeon, this is more than a historical curiosity. Every unnecessary extraction, root-canal treatment, implant-related procedure, or prolonged dental workup may represent a delay in correct imaging, specialist referral, and definitive pain management. Our findings therefore reinforce the need for structured cross-specialty referral criteria between dentistry, oral medicine, neurology, pain medicine, and neurosurgery.

The MRI findings also require careful interpretation. Neurovascular contact was common, but vascular contact alone is not synonymous with symptomatic neurovascular compression, and incidental contact can be seen in asymptomatic individuals. Conversely, the absence of vascular-conflict language in routine documentation does not exclude clinically relevant compression, particularly when outside imaging was unavailable, MRI protocols were incomplete, thin-slice cranial-nerve sequences were not performed, or the relevant finding was subtle. The present study did not include central neuroradiological review and could not assess MRI quality, laterality concordance, degree of nerve distortion, porus trigeminus compression, or short/asymmetric cisternal nerve anatomy. For this reason, MRI variables in this study should be understood as real-world documentation signals rather than definitive imaging biomarkers.

Medication exposure in this cohort underscores how burdensome the pre-procedural pathway can become. More than 80% of patients received carbamazepine, nearly half received pregabalin, one-third received gabapentin, and a quarter received oxcarbazepine. These data fit well with guideline-based care, but they also suggest a substantial burden of stepwise escalation and rescue treatment rather than rapid stabilization on a single first-line medication [3,30,31,32]. In practice, the tertiary trigeminal pain population is often defined as much by what has already failed as by the phenotype itself. This is especially relevant when evaluating candidacy for invasive treatment, because the number and tolerability of prior medication trials frequently shape both patient expectations and physician decision-making.

Our results also provide a useful perspective on procedural sequencing. Procedure use was high across all phenotypes, indicating that tertiary referral tends to concentrate patients who have already crossed the threshold from purely medical management into invasive or semi-invasive care. However, the distribution of procedures was not uniform. Radiofrequency procedures and radiosurgery were used disproportionately in the secondary group, whereas MVD was distributed more evenly. This pattern is consistent with the broader neurosurgical literature, in which MVD remains the most durable option for appropriately selected classical TN, whereas radiosurgery and percutaneous strategies are often favored when anatomy, age, prior treatment, or underlying disease make decompression less attractive [33,34,35,36,37,38,39].

An additional strength of the present cohort is that it captures patients with mixed or non-canonical descriptions, including those with continuous pain. In formal registry studies, such patients are often underrepresented or excluded. In real practice, however, they are common. Our finding that continuous/background pain descriptors were present in approximately two-thirds of the cohort is therefore not surprising; it reflects the fact that tertiary referral enriches for difficult, atypical, recurrent, or partially treated patients rather than pristine textbook cases. This point matters because the coexistence of paroxysmal and continuous pain may signal a more complex pain biology and may partly explain the high recurrence burden observed here [7].

The present study should also be interpreted with appropriate caution. It is retrospective, single-center, derived from routine free text, and based on rule-based NLP-assisted extraction rather than a prospectively curated pain registry. Documentation density was not uniform, and the absence of a documented variable should not be equated with true clinical absence. A blinded dual-review adjudication with formal inter-rater agreement was performed across the entire cohort, and centralized neuroradiological re-review was available. As a result, the imaging and outcome variables should be interpreted as documentation-derived signals.

This limitation is particularly important for complete pain relief and recurrence. These endpoints were extracted from free-text clinical documentation and may compress heterogeneous clinical states, including medication-responsive pain, temporary post-procedural pain freedom, tumor-directed treatment response, repeated procedures, or recurrent symptoms after an initial response. Standardized prospective criteria such as Barrow Neurological Institute pain intensity scores, medication-free remission, time-to-recurrence, and pain-free survival were not consistently available. Therefore, the study is best understood as a real-world pathway and phenotyping analysis rather than a comparative treatment-efficacy study. Nonetheless, the consistency of the extracted patterns and their alignment with contemporary literature support the overall clinical message: tertiary trigeminal pain care is fundamentally a phenotyping problem, and outcomes are inseparable from the diagnostic route by which patients arrive at treatment.

## 5. Conclusions

A large tertiary trigeminal pain cohort revealed marked diagnostic heterogeneity, with only 44.2% of patients falling into a primary/classical TN phenotype and the remainder distributed between secondary disease and dental-first or mimic pathways. The cohort was characterized by heavy medication exposure, frequent procedural escalation, and substantial recurrence documentation. These findings support a structured clinical workflow in which trigeminal-region pain is phenotyped early, secondary causes are actively sought, dental misdirection is recognized explicitly, and procedure selection is tailored to the underlying diagnostic category rather than to pain severity alone. Because imaging and outcome variables were derived from retrospective documentation rather than centralized imaging review or standardized prospective pain scales, these results should be interpreted as real-world pathway signals rather than definitive comparative efficacy estimates.

## Figures and Tables

**Table 1:** Baseline characteristics of the analytic trigeminal pain cohort.

Variable	Value
Source dataset	18,007 note fragments linked to 672 unique patient records
Study period	12 October 2010 to 21 April 2026
Analytic trigeminal pain cohort	455 patients
Explicit TN terminology	311 (68.4%)
Age recoverable	429 (94.3%)
Mean age	58.7 years
Median age	60 years
Sex recoverable	428 (94.1%)
Female sex	267/428 (62.4%)
Branch involvement recoverable	351 (77.1%)
V1 involvement	138 (30.3%)
V2 involvement	256 (56.3%)
V3 involvement	218 (47.9%)
Primary/classical TN	201 (44.2%)
Secondary TN/trigeminal pain	146 (32.1%)
Dental-first or mimic pathway	108 (23.7%)
MRI documented	384 (84.4%)
MRI not explicitly documented in available text	71 (15.6%)
Neurovascular conflict/vascular loop documented	253 (55.6%)
Multiple-sclerosis-related disease	69 (15.2%)
Tumor-related trigeminal involvement	84 (18.5%)
Prior dental evaluation	169 (37.1%)
Prior dental procedure	114 (25.1%)

**Table 2:** Phenotype-stratified pathway characteristics.

Variable	Primary/Classical TN (*n* = 201)	Secondary TN/Trigeminal Pain (*n* = 146)	Dental-First/Mimic Pathway (*n* = 108)	*p* Value
Mean age, years	61.0	57.2	56.9	0.014
Female sex	111 (55.2%)	92 (63.0%)	64 (59.3%)	0.34
MRI documented	164 (81.6%)	130 (89.0%)	90 (83.3%)	0.16
Neurovascular conflict documented	102 (50.7%)	86 (58.9%)	65 (60.2%)	0.18
Prior dental evaluation	0 (0.0%)	61 (41.8%)	108 (100.0%)	<0.001
Prior dental procedure	7 (3.5%)	36 (24.7%)	71 (65.7%)	<0.001
Any invasive/image-guided procedure	167 (83.1%)	134 (91.8%)	91 (84.3%)	0.055
Radiofrequency procedure	49 (24.4%)	53 (36.3%)	24 (22.2%)	0.017
Balloon compression	41 (20.4%)	30 (20.5%)	19 (17.6%)	0.81
Microvascular decompression	51 (25.4%)	34 (23.3%)	28 (25.9%)	0.87
Stereotactic radiosurgery	15 (7.5%)	29 (19.9%)	11 (10.2%)	0.002
Documented complete pain relief	67 (33.3%)	68 (46.6%)	30 (27.8%)	0.004
Documented recurrence	106 (52.7%)	103 (70.5%)	74 (68.5%)	0.001

**Table 3:** Medication and procedural burden in the full cohort.

Variable	*n* (%)
Carbamazepine exposure	367 (80.7%)
Pregabalin exposure	221 (48.6%)
Gabapentin exposure	150 (33.0%)
Oxcarbazepine exposure	116 (25.5%)
Phenytoin exposure	73 (16.0%)
Baclofen exposure	38 (8.4%)
Any invasive/image-guided procedure	390 (85.7%)
Nerve block/injection	355 (78.0%)
Radiofrequency procedure	126 (27.7%)
Balloon compression	90 (19.8%)
Microvascular decompression	113 (24.8%)
Stereotactic radiosurgery	55 (12.1%)
Documented improvement	375 (82.4%)
Documented complete pain relief	165 (36.3%)
Documented recurrence	283 (62.2%)

## Data Availability

The data supporting the findings of this study are presented within the article. Additional de-identified data may be made available from the corresponding author upon request and subject to institutional and ethics approvals, where applicable.
